# Identification of Clinical and Tumor Microenvironment Characteristics of Hypoxia-Related Risk Signature in Lung Adenocarcinoma

**DOI:** 10.3389/fmolb.2021.757421

**Published:** 2021-11-15

**Authors:** Zili Dai, Taisheng Liu, Guihong Liu, Zhen Deng, Peng Yu, Baiyao Wang, Bohong Cen, Liyi Guo, Jian Zhang

**Affiliations:** ^1^ Department of Radiation Oncology, Affiliated Cancer Hospital and Institute of Guangzhou Medical University, State Key Laboratory of Respiratory Diseases, Institute of Respiratory Disease, Guangzhou, China; ^2^ Department of Thoracic Surgery, Affiliated Cancer Hospital and Institute of Guangzhou Medical University, Guangzhou, China; ^3^ Department of Radiation Oncology, DongGuan Tungwah Hospital, Dongguan, China; ^4^ Department of Radiation Oncology, Huizhou Municipal Central Hospital, Huizhou, China; ^5^ Department of Oncology and Hematology, The Six People’s Hospital of Huizhou City, Huiyang Hospital Affiliated to Southern Medical University, Huizhou, China

**Keywords:** lung cancer, hypoxia, immunity, overall survival, risk signature

## Abstract

**Background:** Lung cancer is the leading cause of cancer-related death globally. Hypoxia can suppress the activation of the tumor microenvironment (TME), which contributes to distant metastasis. However, the role of hypoxia-mediated TME in predicting the diagnosis and prognosis of lung adenocarcinoma (LUAD) patients remains unclear.

**Methods:** Both RNA and clinical data from the LUAD cohort were downloaded from the Cancer Genome Atlas (TCGA) and Gene Expression Omnibus (GEO) databases. Both univariate and multivariate Cox regression analyses were used to further screen prognosis-related hypoxia gene clusters. Time-dependent receiver operation characteristic (ROC) curves were established to evaluate the predictive sensitivity and specificity of the hypoxia-related risk signature. The characterization of gene set enrichment analysis (GSEA) and TME immune cell infiltration were further explored to identify hypoxia-related immune infiltration.

**Results:** Eight hypoxia-related genes (LDHA, DCN, PGK1, PFKP, FBP1, LOX, ENO3, and CXCR4) were identified and established to construct a hypoxia-related risk signature. The high-risk group showed a poor overall survival compared to that of the low-risk group in the TCGA and GSE68465 cohorts (p < 0.0001). The AUCs for 1-, 3-, and 5-year overall survival were 0.736 vs. 0.741, 0.656 vs. 0.737, and 0.628 vs. 0.649, respectively. The high-risk group was associated with immunosuppression in the TME.

**Conclusion:** The hypoxia-related risk signature may represent an independent biomarker that can differentiate the characteristics of TME immune cell infiltration and predict the prognosis of LUAD.

## Introduction

Lung cancer is the most common malignant tumor and one of the leading causes of cancer-related death worldwide (Sung et al., 2021). Non-small cell lung cancer (NSCLC) accounts for approximately 85% of lung cancer cases, which comprises approximately 40–50% cases of lung adenocarcinoma (LUAD) and 20–30% cases of lung squamous cell carcinoma (LUSC) (Liu et al., 2021; Siegel et al., 2020). Despite advances in chemoradiotherapy and targeted therapies, immune checkpoint inhibitors (ICI), including programmed death-ligand 1 (PD-L1), programmed cell death 1 (PD-1), and cytotoxic T lymphocyte antigen-4 (CTLA-4) represent promising advances in the treatment of lung cancer (Hiley et al., 2016; Bhandari et al., 2021; Fountzilas et al., 2021); however, the clinical response rate of ICIs is only 20%, which can seriously hinder its wider application (Borghaei et al., 2015; Brahmer et al., 2015; Reck et al., 2016; Rittmeyer et al., 2017).

Increasing evidence suggests that the accumulation of immunosuppressive cell subsets within the tumor microenvironment (TME) (e.g., tumor-associated macrophages [TAM], myeloid-derived suppressor cells [MDSCs], and regulatory T cells [Tregs]) can influence the prognosis and clinical benefit of ICI therapy (Chen et al., 2021; Fogli et al., 2021; Semba et al., 2021). Multiple immunosuppressive mechanisms in the TME, including the tumor mutation burden (TMB), PD-L1 expression and tumor-infiltrating lymphocytes (TILs), have been identified as major factors that regulate immune resistance (Ji et al., 2012). As a feature of unstable vasculature and a high metabolic rate, hypoxia is a hallmark of tumorigenesis in various cancers (Semenza, 2014). Hypoxia can both induce an immunosuppressive TME, which decreases the effect of immunotherapy (Fukumura et al., 2018), as well as upregulate PD-L1 expression, which further promotes tumor escape (Barsoum et al., 2014; Noman et al., 2014; Koh et al., 2016; Ruf et al., 2016). The hypoxia-related gene signature may be a key regulator in mediating tumor immune evasion. Thus, the identification of a hypoxia-related risk signature may predict the subpopulations of clinical ICI therapy and provide a novel means of improving the clinical curative effect.

In this study, mRNA expression and the clinical information of LUAD samples were downloaded from the TCGA and GEO databases. Eight hypoxia-related genes were identified and established to construct a hypoxia-related risk signature. The risk signature could differentiate the high- and low-risk subgroups, and a high-risk hypoxia signature has been associated with the inactivation of TME immune cell infiltration. Thus, targeting hypoxia-related genes may represent novel therapeutic targets that can enhance the proportion of LUAD patients who can be treated with ICIs.

## Methods

### Data Acquisition and Processing

RNA expression and clinical data related to the LUAD cohort were downloaded from The Cancer Genome Atlas (TCGA, http://cancergenome.nih.gov/) and GENE EXPRESSION OMNIBUS database (GEO, https://www.ncbi.nlm.nih.gov/geo/). The independent cohort was used to verify the results of the TCGA dataset. Two authors (ZLD and TXL) independently reviewed the RNA-seq transcriptome and clinical data from both datasets to avoid any potential errors.

### Construction of a Protein-Protein Interaction Network

To identify hypoxia-related hub genes, a protein-protein interaction (PPI) network was constructed using the STRING database (http://string-db.org). Genes with a node degree >0.4 were considered to be hub genes in the PPI network. PPI network visualization and analysis were further performed using Cytoscape software (https://cytoscape.org/).

### Establishment of a Hypoxia-Related Risk Signature

To establish the hypoxia-related risk signature, a univariate Cox regression analysis was used to screen for prognosis-related hypoxia-associated genes. A multivariate Cox regression analysis was further used to calculate the corresponding risk coefficient according to the gene expression of the input gene set, and the risk score was created for each patient. The risk score was calculated using the following formula:

$$\text{risk}\;\text{score}=\sum_{i=1}^{n}\left({\text{Exp}}_{i}*{\text{Coe}}_{i}\right)$$

where Exp_i_ represents the level of hypoxia gene expression, and Coe_i_ represents the corresponding multivariate Cox regression coefficient.

### Gene Set Enrichment Analysis

Patients were divided into low- and high-risk groups based on the median risk score. A gene set enrichment analysis (GSEA) 3.0 (http://www.broad.mit.edu/gsea/) detected different signaling genes. Each analysis performed 1,000 gene combinations. NES >1 and nominal *p* < 0.05 were considered to be statistically significant.

### Development of Receiver Operating Characteristic Curves

To assess the hypoxia related risk signature, a univariate Cox regression was used to analyze prognostic hub genes with clinical information. Significant prognostic hub genes were further analyzed using a multivariate Cox regression analysis. A receiver operating characteristic (ROC) analysis was performed to determine the sensitivity and specificity of the risk model for predicting the OS.

### Evaluation of Immune Cell Type Factions

To characterize the immune cell types in the TME, CIBERSORT (https://cibersort.stanford.edu/) was used to clarify the deconvolution of the immune cell subtype expression matrix based on linear support vector regression. In accordance with the methods described by Zhang J. et al. (2020), the immune infiltration characteristics of 22 immune cell subpopulations were evaluated between high- and low-related risk groups in LUAD.

### Statistical Analysis

Statistical analyses were performed using standard R packages (version 3.6.2). A Student’s t-test was used to compare the continuous and discrete variables. A Pearson’s chi-squared test was used to compare the categorical clinicopathological variables. The Kaplan-Meier method was used to assess the OS and differences were assessed using a two-sided log-rank test. *p* < 0.05 indicated statistical significance.

## Results

### Establishment of a Hypoxia-Related Risk Signature

**FIGURE 1:**
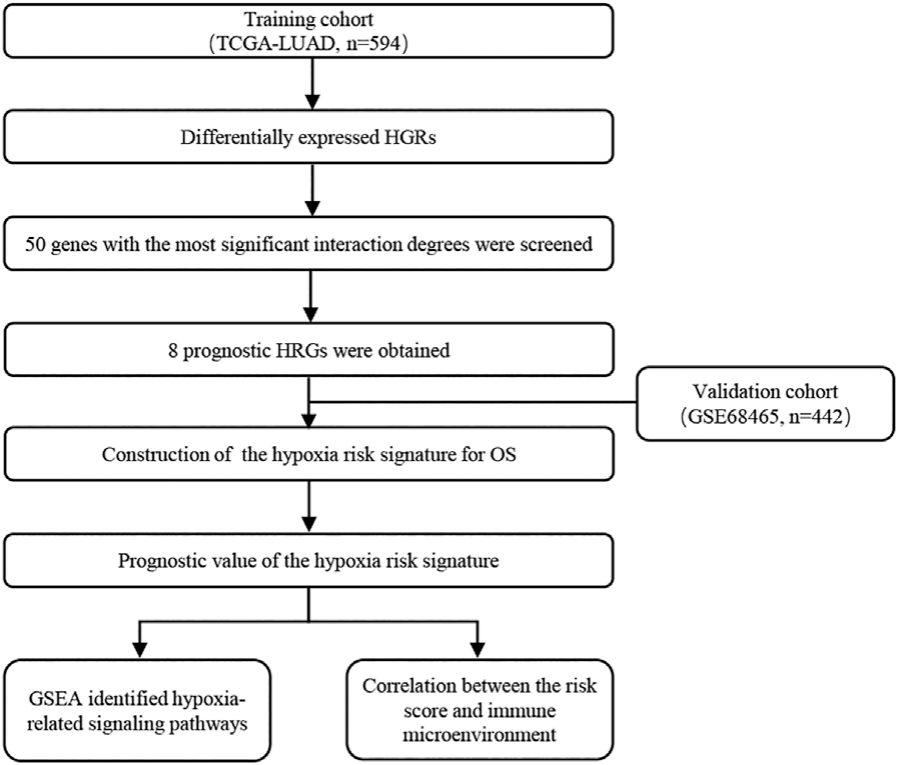
The work flow of this study.

**FIGURE 2:**
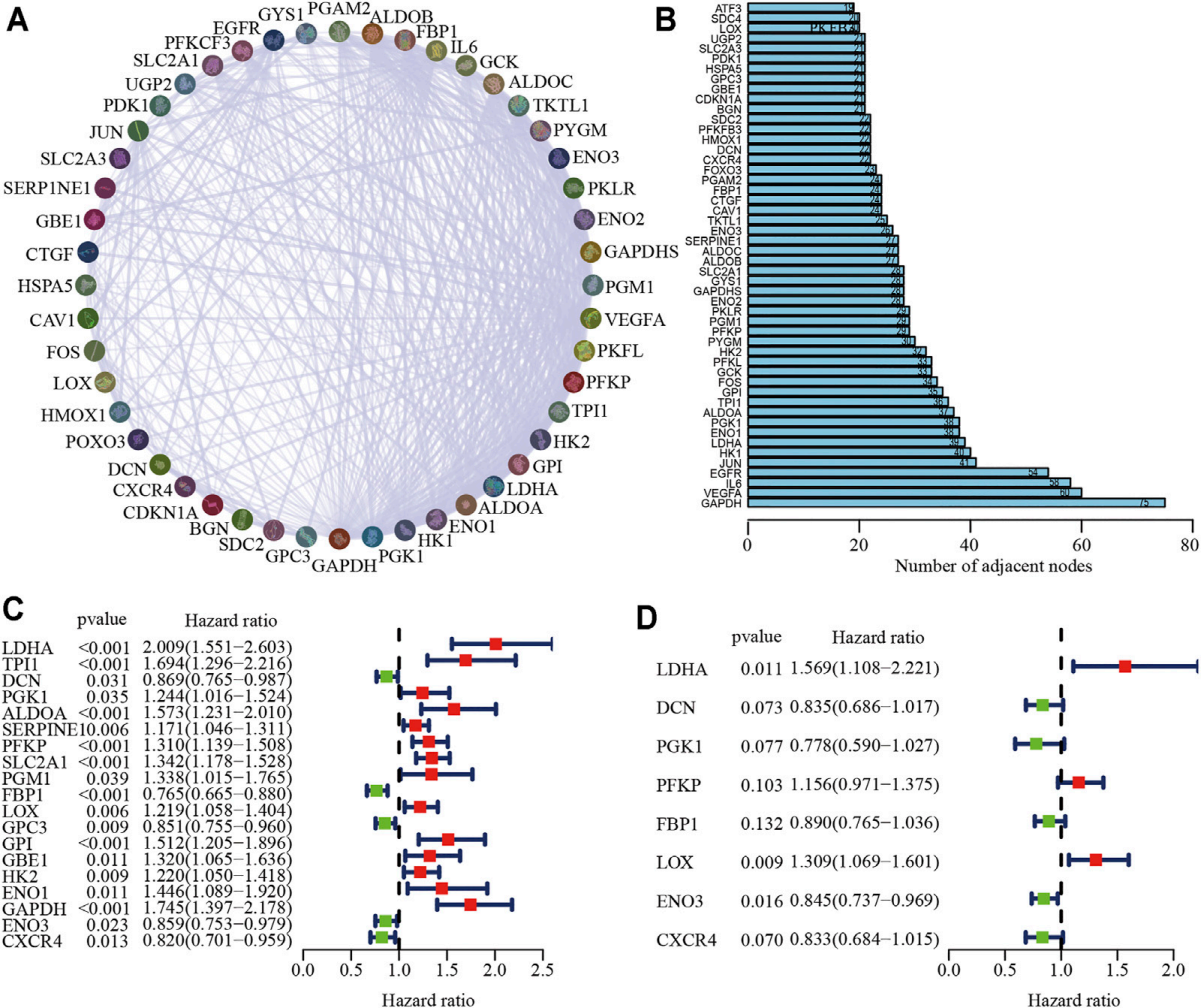
Identification of key hypoxia-related genes in LUAD. **(A)** The protein-protein interaction network of hypoxia-related genes. **(B)** A total of 50 of the most relevant hypoxia-related key genes. **(C, D)** Univariate **(C)** and multivariate Cox **(D)** regression analysis of key hypoxia-related genes.

Details of the clinical data from the two cohorts used in this study are listed in Supplementary Table S1. Figure 1 shows the flow chart of the process used to screen hypoxia-related genes, and the hypoxia-related gene set was downloaded from the TCGA-LUAD cohort. To investigate the interactive roles of hypoxia-related genes, a PPI network analysis was applied using the STRING online database and Cytoscape software (Figure 2A). The 50 genes with the most significant interactions were obtained (Figure 2B). A univariate Cox regression analysis revealed that 19 key genes were significantly associated with overall survival (OS) in patients with LUAD (*p* < 0.05; Figure 2C). A multivariate Cox regression analysis further showed that eight hypoxia-related genes, including lactate dehydrogenase A (LDHA), decorin (DCN), phosphoglycerate kinase 1 (PGK1), phosphofructokinase (PFKP), fructose-bisphosphatase 1 (FBP1), lysyl oxidase (LOX), enolase 3 (ENO3), and C-X-C Motif Chemokine Receptor 4 (CXCR4), were obtained (Figure 2D). The correlation analysis showed that there was a significant correlation among the hypoxia-related genes in the TCGA-LUAD and GSE68465 cohort, including a positive correlation for DCN and CXCR4, and a negative correlation for DCN and LDHA (Supplementary Figure S1). The hypoxia-related risk signature was developed based on the key eight hypoxia-related genes. The risk score formula was listed as follows: hypoxia related risk signature = (0.45 × LDHA) + (−0.18×DCN) + (−0.25 × PGK1) + (0.14 × PFKP) + (−0.12 × FBP1) + (0.27 × LOX) + (−0.17 × ENO3) + (−0.18 × CXCR4).

### Prognostic Significance of the Hypoxia Risk Signature

**FIGURE 3:**
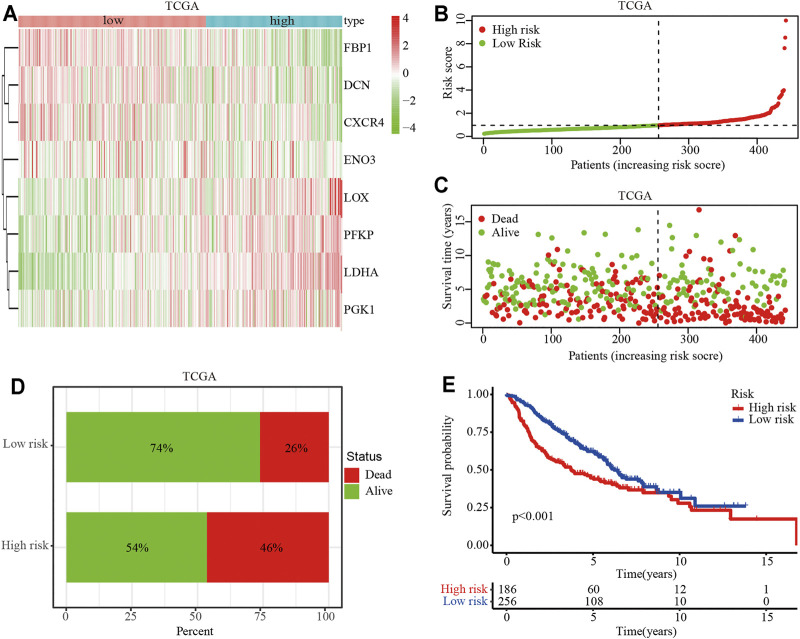
Prognostic value of the hypoxia-related risk signature in the TCGA database. **(A)** Heatmap of high- and low-risk LUAD patients stratified by eight key hypoxia-related genes in the TCGA cohort. **(B)** Distribution and median survival time of the high- and low-risk LUAD patients in the TCGA cohort. **(C)** Distribution of the survival status of high- and low-risk LUAD patients. The *X*-axis represents the number of patients, and the *Y*-axis represents the survival time. **(D)** Survival and mortality rates in the high- and low-risk LUAD patients. **(E)** OS analysis of high- or low-risk LUAD patients.

**FIGURE 4:**
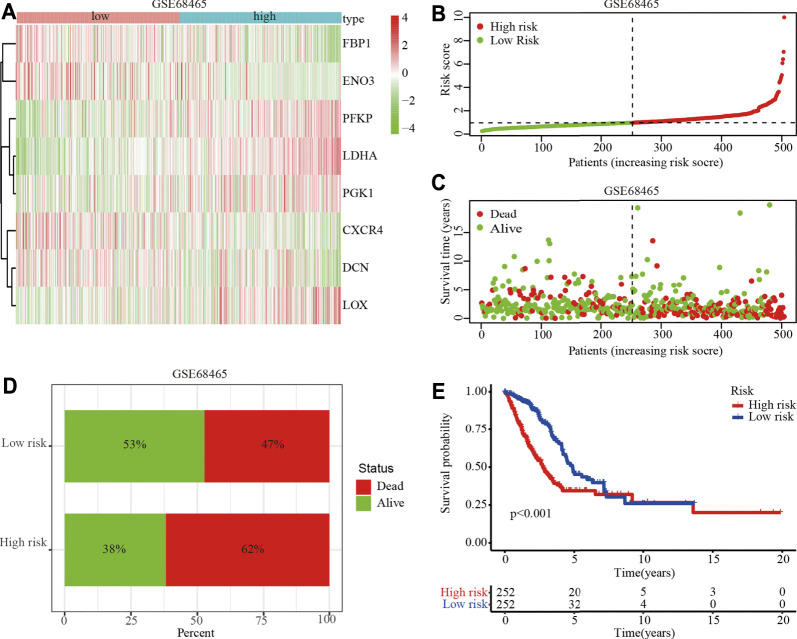
Prognostic value of the hypoxia-related risk signature in the GSE68465 dataset. **(A)** Heatmap of high- and low-risk LUAD patients stratified by eight hypoxia related key genes in GSE68465 dataset **(B)** Distribution and median survival time of high- and low-risk LUAD patients in the GSE68465 dataset. **(C)** Distribution of high- and low-risk LUAD patients. **(D)** Survival and mortality in the high- and low-risk LUAD patients. **(E)** OS analysis of high- or low-risk LUAD patients.

To identify the clinical application of the hypoxia-related risk signature, a cluster analysis showed that the TCGA-LUAD cohort could be divided into high- (*n* = 297) and low-risk (*n* = 297) groups and differential expression was observed in the hypoxia-related genes between the two groups (Figure 3A). The distribution of risk scores and survival status of the TCGA-LUAD patients are shown in Figures 3B,C. The further percentage of survival showed that compared with the 26% death in the low-risk group, the high-risk group had a 46% death (Figure 3D). A prognostic analysis identified that the high risk group had a poor survival compared to the low risk group in the TCGA-LUAD cohort (*p* < 0.001; Figure 3D). The hypoxia-related risk signature was further validated in the GSE68465 cohort. A total of 442 patients were stratified into high- (*n* = 221) and low-risk (*n* = 221) subgroups using the median risk score values (Figure 4A). The LUAD patients in the high-risk group exhibited a higher probability of earlier death (Figures 4B,C) and had significantly worse OS compared to those in the low-risk group (*p* < 0.001; Figure 4D). Taken together, these results indicate that the hypoxia-related risk signature may function as a biomarker to predict the prognosis of patients with LUAD.

### Evaluation Value of the Hypoxia Risk Signature

**FIGURE 5:**
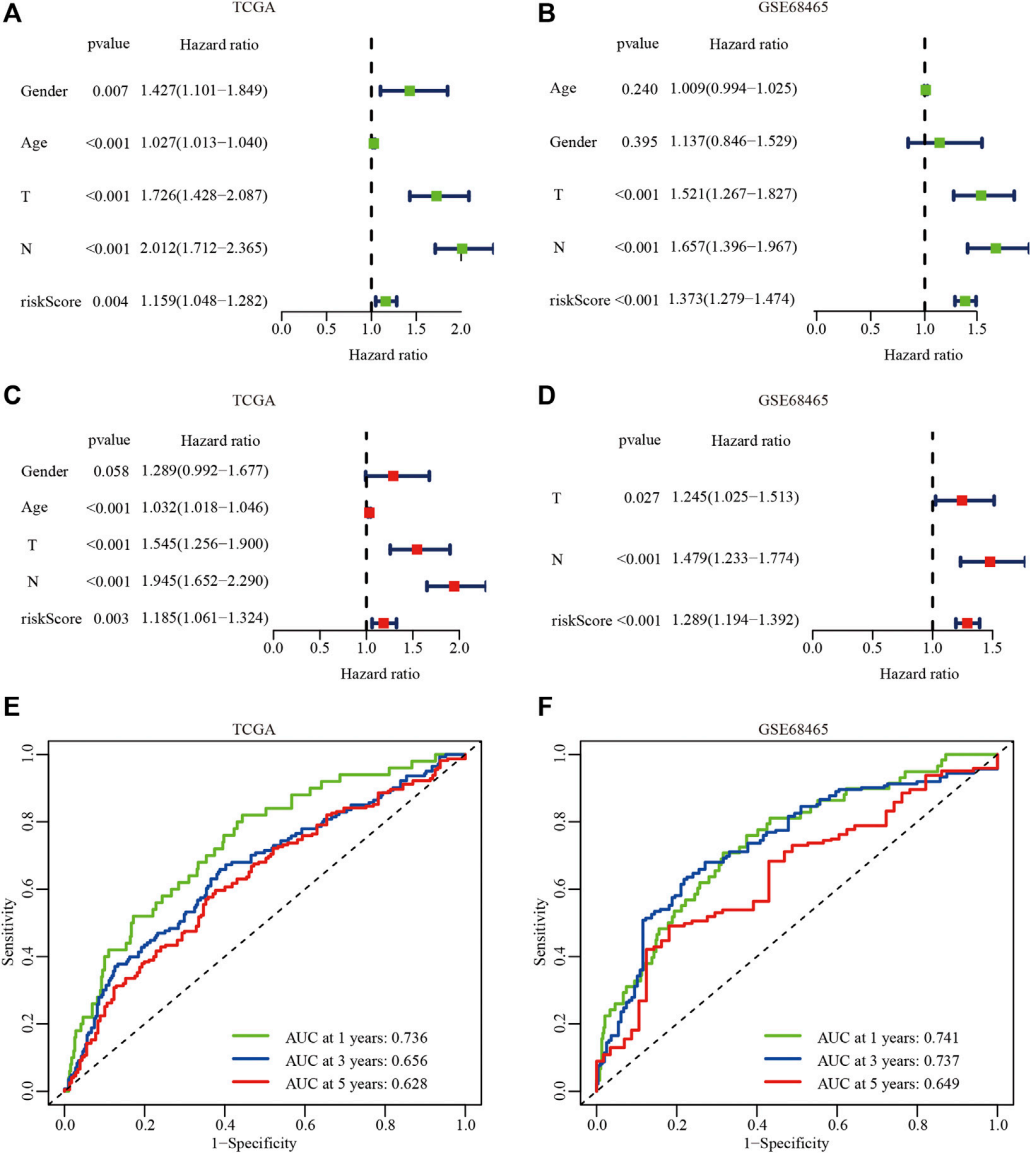
The independent prognostic analysis of the hypoxiarelated risk signature. **(A, B)** Univariate Cox analysis of clinical characteristics and hypoxiarelated risk signature in the TCGA **(A)** and GSE68465 dataset **(B)**. **(C, D)** Multivariate Cox analysis in the TCGA **(C)** and GSE68465 dataset **(D)**. **(E, F)** AUC of the hypoxia risk signature in the TCGA **(E)** and GSE68465 datasets **(F)**.

To determine whether the hypoxia-related risk signature could be used as an independent prognostic factor, univariate, and multivariate Cox regression analyses were performed to evaluate the signature-based risk score using the TCGA and GSE68465 cohorts. The results of the univariate Cox regression analysis indicated that tumor (T) stage, lymph node (N) stage, and hypoxia-related risk score were positively correlated with the OS in the TCGA and GSE68465 cohorts (*p* < 0.001; Figures 5A,B). The multivariate survival analysis showed that the T stage, N stage, and hypoxia-related risk score were significantly associated with the OS, which suggested that the hypoxia-related risk score could be defined as an independent prognostic factor in patients with LUAD (*p* < 0.001; Figures 5C,D).

To further evaluate the predictive accuracy of the hypoxia-related risk signature, the ROC curves of the hypoxia-related risk signature were performed. The results showed that AUC at 1, 3, and 5 years in the TCGA-LUAD and GSE68465 cohorts were 0.736 *vs* 0.741, 0.656 *vs* 0.737, and 0.628 *vs* 0.649, respectively (Figures 5E,F). These results suggest that the hypoxia-related risk signature had an excellent predictive prognostic ability and provided a useful biomarker with clinical application.

### Immune Cell Infiltration in the Tumor Microenvironment (TME)

A GSEA analysis was performed to investigate the potential signaling pathways activated by hypoxia-related genes, The results showed that the hypoxia-related genes were associated with interferon gamma, B cell, and natural killer (NK0 cell infiltration in the TCGA and GSE68465 cohorts (Supplementary Figure S2). These results indicate that the hypoxia-related genes mediated the malignant features of LUAD by regulating immune cell infiltration in the TME.

**FIGURE 6:**
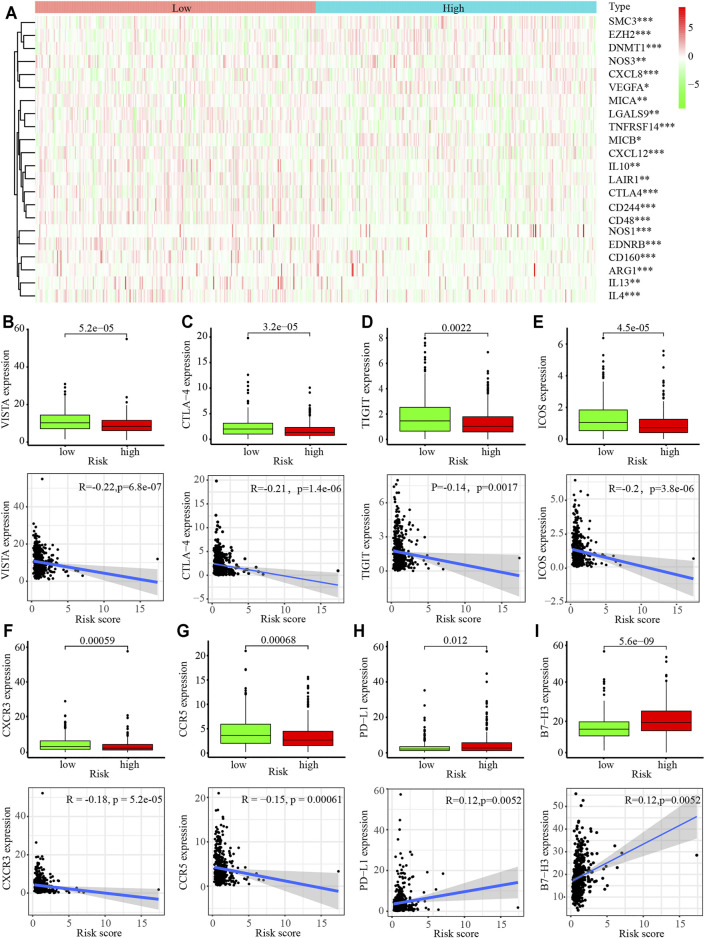
Immune cell infiltration in the tumor microenvironment (TME) in LUAD. **(A)** Heatmap of immune-related gene sets between the high- and low-risk LUAD patients. **(B–G)** Expression of VISTA **(B)**, CTLA4 **(C)**, TIGT **(D)**, ICOS **(E)**, CXCR3 **(F)**, CCR5 **(G)** PD-L1 **(H)**, and B7-H3 **(I)** in high- and low-risk LUAD patients.

Thus, we next analyzed the immune cell infiltration of the 22 immune cell subgroups using the CIBERSORT algorithm. The results showed that the distribution ratio of the infiltrating immune cells between the high- and low-risk groups in the TCGA and GSE68465 cohorts were significantly different (Supplementary Figures S3A,B). A component analysis of the immune cells from the TME showed that activated CD4^+^ T memory cells, resting NK cells, M0 and M1 macrophages, resting mast cells, and resting dendritic cells were significantly different between the high- and low-risk groups (*p* < 0.05; Supplementary Figures S3C–H). A cluster analysis revealed that there was a distinct difference in the immune molecules between the high- and low-risk groups (*p* < 0.05; Figure 6A). The relative expression analysis indicated that the expression of V-domain Ig Suppressor of T cell Activation (VISTA), cytotoxic T-lymphocyte associated protein 4 (CTLA4), T cell immunoreceptor with Ig and ITIM domains (TIGIT), inducible T cell costimulator (ICOS), C-X-C motif chemokine receptor 3 (CXCR3), and C-C motif chemokine receptor 5 (CCR5) were significantly downregulated in the high-risk groups (*p* < 0.05; Figures 6B–G). In contrast, PD-L1 and B7-H3 expression were significantly upregulated in the high-risk groups (*p* < 0.05; Figures 6H,I). A correlation analysis revealed that VISTA, CTLA4, TIGIT, ICOS, CXCR3, and CCR5 were negatively associated with the hypoxia-related risk score, whereas PD-L1 and B7-H3 were positively associated with the hypoxia-related risk score (*p* < 0.05; Figures 6B–I). These results revealed that the hypoxia-related risk signature may be involved in tumorigenesis by regulating immune cell infiltration into the TME and can be used to predict patient prognosis.

## Discussion

Lung cancer is the leading cause of cancer-related death worldwide (Bray et al., 2018; Siegel et al., 2020). Chemoradiotherapy and targeted therapies are the dominant therapeutic strategies used to treat patients with NSCLC; however, the prognosis is poor, with a median OS of only 9–11 months (Scagliotti et al., 2008; Sun et al., 2016). Hypoxia is an important phenomenon associated with solid tumors that contributes to metastasis, deregulation of the tumor microenvironment (TME), and resistance to therapy (Goyette et al., 2021). In this study, we comprehensively analyzed the expression of hypoxia-related genes in the TCGA and GEO databases, and established a hypoxia-related risk signature, which can differentiate LUAD patients into high- and low-risk groups.

The hypoxia-related risk signature consists of eight hypoxia-related genes, including LDHA, DCN, PGK1, PFKP, FBP1, LOX, ENO3, and CXCR4. LDHA is an enzyme that catalyzes the mutual conversion of pyruvate and lactic acid, as well as promotes invasion, metastasis, nest loss, and apoptosis resistance in various cancers (Cheng et al., 2021; Crowley et al., 2021; Gupta et al., 2021). Multiple studies have suggested that DCN can suppress lung cancer progression by blocking receptor tyrosine kinases (Horvath et al., 2014). Moreover, decreased DCN expression correlates with lymphatic metastasis in patients with lung cancer (Biaoxue et al., 2011). While PGK1 up-regulation was found to trigger autophagy in tumorigenesis (Qian et al., 2017a; Qian et al., 2017), it was also associated with resistance to chemoradiotherapy (Cai et al., 2019; Sun et al., 2015). PFKP is a rate-limiting enzyme involved in glycolysis that has been found to be upregulated in various types of cancer (Park et al., 2013; Wang et al., 2016; Kim et al., 2017). NK cell dysfunction induced by FBP1 inhibited glycolysis during lung cancer progression (Cong et al., 2018). LOX upregulation in cancer has been shown to be involved in cancer progression and metastasis (Murdocca et al., 2021). A knockdown of ENO3 expression exhibited a selective anticancer effect in STK11 mutant lung cancer cells (Park et al., 2019). In addition, a CXCR4 blockade can improve anti-PD-L1 therapy in triple negative breast cancer(Zhou et al., 2021). These results indicate that targeting hypoxia-related risk genes may represent a promising method of treating patients with lung cancer.

In this study, a multivariate Cox regression analysis of the hypoxia-related risk signature, which could indicated that it could act as an independent predictor of OS in LUAD. The predictive prognostic value of hypoxia-related risk signatures is greatly validated in TCGA and GEO database. However, the signatures need to be validated in prospective studies. GSEA revealed that hypoxia-related genes (LDHA, DCN, PGK1, PFKP, FBP1, LOX, ENO3, and CXCR4) involved in various immune cell infiltration in the TME. The CIBERSORT analysis also identified distinct differences in the distribution of immune cells between the high- and low-risk groups. The correlation analysis also verified that the expression of immune check-point molecules, including PD-L1, were associated with the hypoxia-related risk score. Recent studies have identified that FOXO4 regulated the glycolysis process of gastric cancer by disrupting the HIF-1α-FOXO4-LDHA axis (Wang et al., 2021). Hypoxia-induced circular RNA has_circRNA_403,658 promotes bladder cancer cell growth through activation of LDHA (Wei et al., 2019). Activation of PGK1 under hypoxic conditions promoted glycolysis and increased stem cell-like properties and the epithelial-mesenchymal transition in oral squamous cell carcinoma cells (Zhang Y. et al., 2020). Hyperbaric oxygen therapy repressed the warburg effect and epithelial-mesenchymal transition in hypoxic NSCLC cells via the HIF-1α/PFKP axis (Zhang et al., 2021). The study by Li et al. reported that a GBE1 blockade promoted the secretion of CCL5 and CXCL10 to recruit CD8^+^ T lymphocytes into the TME and upregulate PD-L1 expression in LUAD cells via the IFN-I/STING signaling pathway (Li et al., 2019). EML4-ALK enhanced PD-L1 expression in LUAD via HIF1α and STAT3 (Koh et al., 2016). Thus, hypoxia-related risk genes may represent a novel target for immunotherapy in LUAD by modulating cell infiltration into the TME.

However, there were also several limitations associated with this study. First, since all the data in this study were derived from public databases and retrospective analyses, the hypoxia-related risk signature must be further validated by multiple centers. Second, TME cell infiltration had a distinct distribution between the high- and low-risk groups; thus, the potential function and mechanisms mediated by the hypoxia-related risk genes must be further explored.

## Conclusion

In summary, we performed a comprehensive genomic analysis of hypoxia-related risk genes and established a hypoxia-related risk signature that could stratify the risk and predict OS in patients with LUAD by modulating TME cell infiltration. Thus, targeting hypoxia-related risk genes may represent a promising method of improving the immunotherapeutic efficiency of patients with lung cancer.

## Data Availability

The original contributions presented in the study are included in the article/Supplementary Material, further inquiries can be directed to the corresponding authors.

## Abbreviations

CTLA-4, cytotoxic T-lymphocyte antigen-4; GSEA, Gene set enrichment analysis; ICI, immune checkpoint inhibitors; LUAD, lung adenocarcinoma; LUSC, lung squamous cell carcinoma; MDSC, myeloid-derived suppressor cells; NSCLC, Non-small cell lung cancer; OS, overall survival; PD-L1, programmed death-ligand 1; PD-1, programmed cell death 1; PPI, protein-protein interaction; ROC, receiver operating characteristic; TME, tumor microenvironment; TAM, tumor-associated macrophages; Treg, regulatory T cells; TMB, tumor mutation burden; TIL, tumor-infiltrating lymphocytes.
