# Supplementary material for: Identification of Clinical and Tumor Microenvironment Characteristics of Hypoxia-Related Risk Signature in Lung Adenocarcinoma
Source: Front Mol Biosci. 2021 Nov 15;8:757421. doi: 10.3389/fmolb.2021.757421 (PMC8634728; doi:10.3389/fmolb.2021.757421)
Supplement: Supplementary file 2 [file Table1.DOCX]

| **Table S1.** Patient baseline characteristics | | |
| --- | --- | --- |
| **Variable** | **Number (N)** | |
|  | **TCGA-LUAD cohort** | **GSE68465 cohort** |
| Age |  |  |
| ＜60 | 139 | 128 |
| ≥ 60 | 383 | 315 |
| Gender |  |  |
| Male | 242 | 220 |
| Female | 280 | 223 |
| Stage |  |  |
| I | 279 | - |
| II | 124 | - |
| III | 85 | - |
| IV | 26 | - |
| T |  |  |
| T1 | 175 | 150 |
| T2 | 281 | 251 |
| T3 | 47 | 28 |
| T4 | 19 | 12 |
| M |  |  |
| M0/MX | 493 | - |
| M1 | 25 | - |
| N |  |  |
| N0/NX | 346 | 300 |
| N1 | 98 | 88 |
| N2 | 75 | 53 |
| N3 | 2 | NA |

NA = not applicable
