# Supplementary figures and images for: Identification of Clinical and Tumor Microenvironment Characteristics of Hypoxia-Related Risk Signature in Lung Adenocarcinoma
Source: Front Mol Biosci. 2021 Nov 15;8:757421. doi: 10.3389/fmolb.2021.757421 (PMC8634728; doi:10.3389/fmolb.2021.757421)

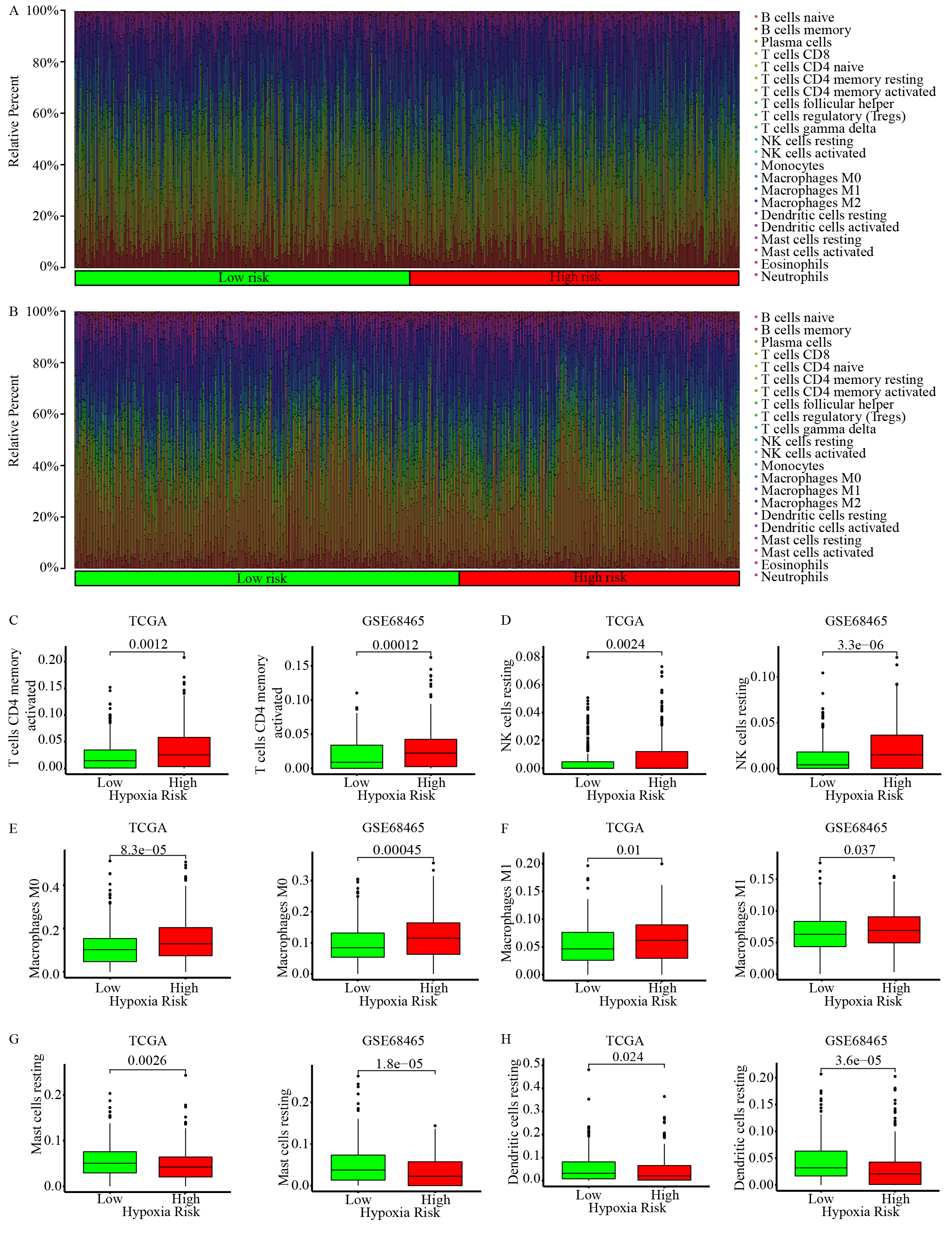

Supplement: Supplementary file 3 [file Image3.TIF]

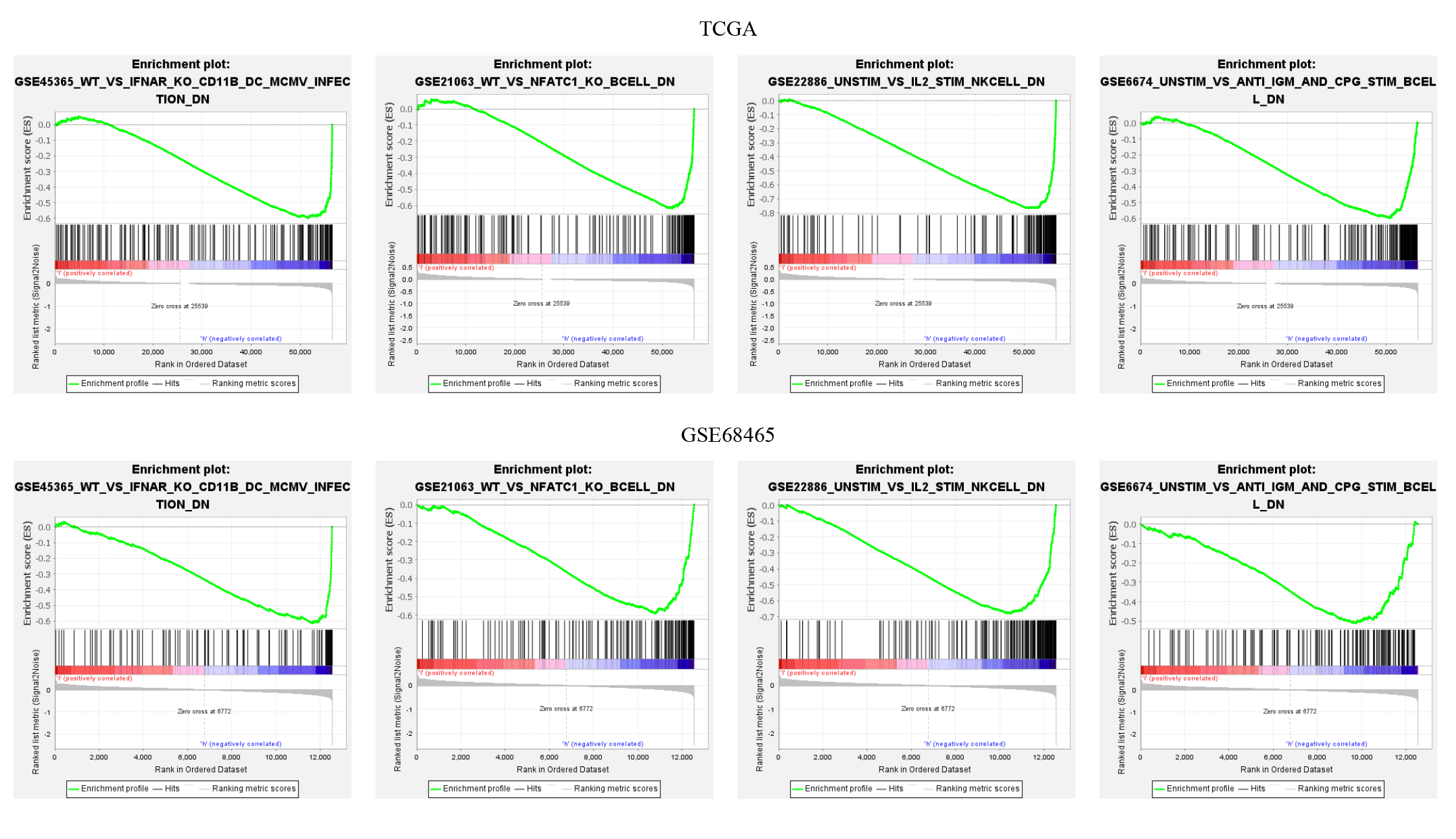

Supplement: Supplementary file 4 [file Image2.TIF]

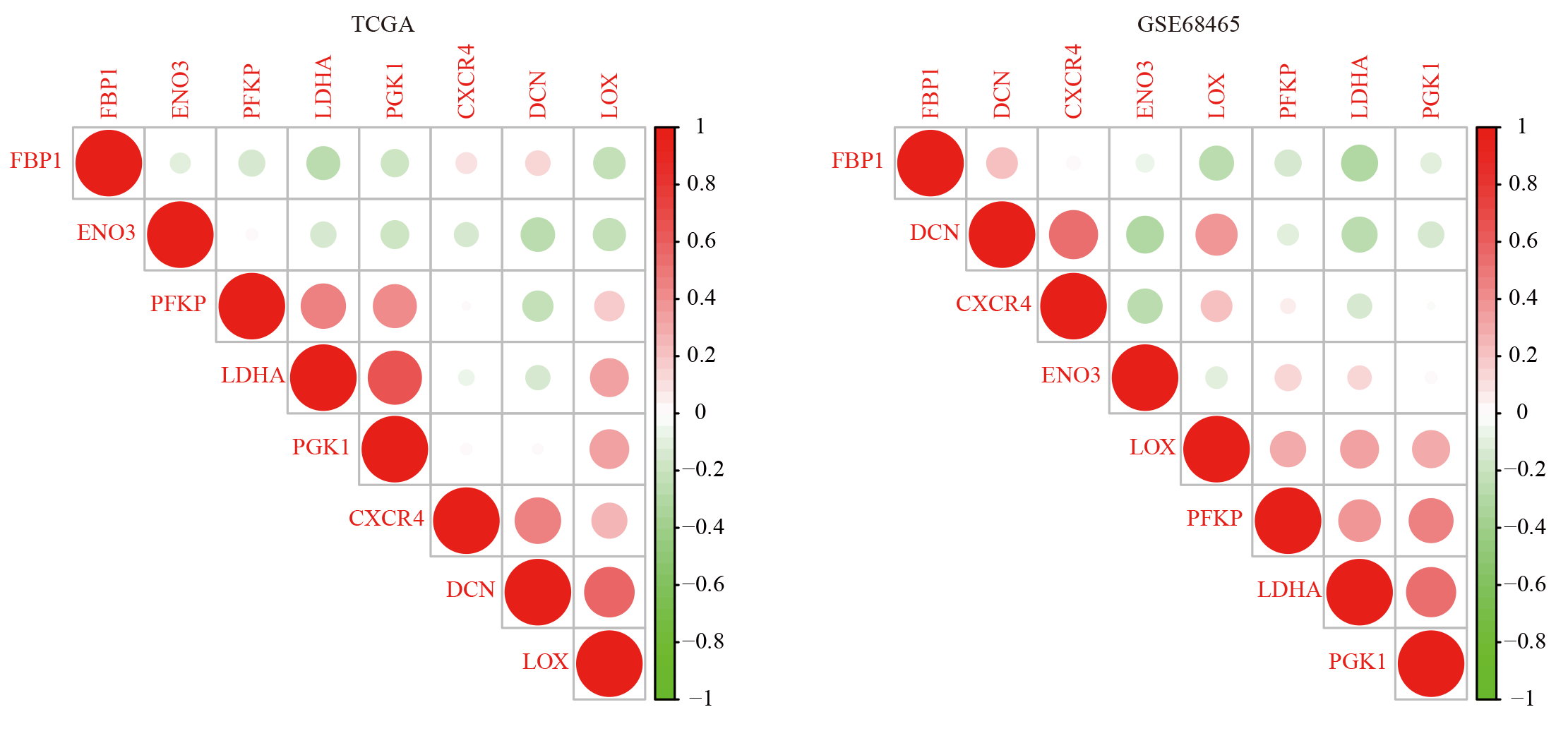

Supplement: Supplementary file 6 [file Image1.TIF]
